# Cost-effective drone monitoring and evaluating toolkits for stream habitat health: development and application

**DOI:** 10.1007/s10661-025-14814-9

**Published:** 2025-12-05

**Authors:** Wei Wang, Boyuan Lu, Chin H. Wu

**Affiliations:** Department of Civil and Environmental Engineering, University of Wisconsin-Madison, Madison, WI 53706 USA

**Keywords:** Unmanned aerial vehicles, Stream habitat health, Multi-metric indices, Cost-effective monitoring, Continuous spatial assessments

## Abstract

Effective monitoring for stream habitat health is essential for sustainable restoration and management to conserve ecosystem services. Traditional in situ surveying with multi-metric indices is widely used by state and federal agencies but is often labor-intensive and spatially limited. To address these challenges, this study introduces a suite of cost-effective drone-based monitoring toolkits that integrate automated flight-route design, Structure-from-Motion (SfM) image reconstruction, and multi-metric index (MMI) computation. The toolkits form an end-to-end workflow for quantitative habitat evaluation, enabling centimeter-scale mapping and standardized MMI scoring based on Wisconsin DNR and EPA guidelines. The toolkits were examined at Black Earth Creek, Wisconsin, where high- and low-elevation drone images were collected and processed to generate high-resolution (< 1 cm) ortho-photos and terrain maps. The reconstructed terrain achieved horizontal and vertical accuracies of 0.04 m and 0.17 m, respectively, and the UAV-derived MMI results showed strong agreement with field measurements. The entire process—from flight planning to assessment—can be completed by one operator within three hours using an off-the-shelf drone (< USD 1000) and standard photogrammetry software. Beyond improving efficiency, the integrated toolkits provide spatially continuous, repeatable assessments that capture habitat variability and degradation hotspots often missed by conventional transect-based methods. By significantly reducing labor and costs associated with data collection, processing, and assessment, these UAV-based toolkits offer a cost-effective, scalable, and reproducible solution for routine stream habitat monitoring and long-term conservation planning.

**Supplementary Information:**

The online version contains supplementary material available at 10.1007/s10661-025-14814-9.

## Introduction

Stream habitat health, defined as the condition of areas supporting aquatic organisms for concealment, breeding, and feeding (Karr, 1999; United States Environmental Protection Agency, 2022), is important for determining the efficacy of aquatic communities (Maddock, 1999). First, stream habitat health is associated with the species density and community composition of riparian vegetation (Hough-Snee, et al., 2013; Nilsson & Svedmark, 2002), as well as aquatic organisms such as fish and macroinvertebrates (Sonkar et al., 2023; Zheng et al., 2023). Second, stream habitat health is essential for providing ecosystem services, such as mitigate flood risk (Darby, 1999), soil conservation (Saad et al., 2018), pollutant assimilation (Dosskey et al., 2010), and recreation (Hughes, 2015). Third, stream habitat health is sensitive to anthropogenic alterations (Allan, 2004; Arthington et al., 2010) such as urbanization, dams, and pollutant discharge (Malmqvist & Rundle, 2002; Roni, et al., 2008) as well as other disturbances like invasive species (Scott & Helfman, 2001) and climate change (Null, et al., 2013). Given these importance and vulnerabilities, effective characterization of stream habitat health requires consistent, science-based monitoring.

The evaluation of stream habitat health, in practice, relies on multi-metric indices (MMIs), which integrate multiple attributes from streams and riparian zones to provide a holistic assessment. Early-stage MMIs, such as an index of biotic integrity (IBI), compare fish populations and species richness under human influence with natural reference conditions (Karr, 1981). Revised MMIs incorporate more physical, chemical, and biological attributes for comprehensive assessments of regional stream habitat. Key attributes include physical habitat (Fausch et al., 1984), water quality (Karr, 1986), biological activity (Fore et al., 1996), and sediment transport (Allan et al., 1997). These MMIs have become a cornerstone for monitoring programs conducted by local and national agencies, as summarized in an EPA report (Barbour, 1999). Recent advancements have expanded MMIs to account for human disturbances relative to unimpaired conditions (Oberdorff et al., 2002; Somerville, 2010) and to be applicable across a broader range of stream types (Bolding et al., 2020; Mamun & An, 2020). As MMIs continue to be developed and refined, their effectiveness hinges on the implementation of reliable and systematic monitoring systems.

Conventional stream monitoring measures physical geometry data such as width, depth, and bed slope using tape measures (Simonson, 1994), and surveys ecological data like biodiversity of species, vegetation covers, and substrates by transect or quadrat sampling (Wang et al., 1996). However, these approaches have several limitations. First, assessments of some descriptors, such as riffles and pools, are subjective and could be affected by accessor bias (Woodget et al., 2016). Second, some locations are difficult to access due to deep or fast-flowing water (Cavanagh et al., 1997). Third, discrete sampling data with limited transects or quadrats can misinterpret the spatial variation of highly diverse landscapes (Cooper et al., 1997). Finally, data collection is labor-intensive and time-consuming (Simonson et al., 1994), especially for larger streams. While traditional methods provide valuable ground truth data, these challenges underscore the need for alternative monitoring approaches that are reliable, safe, continuous, and efficient.

Remote sensing techniques, such as satellite-based imagery, aerial photogrammetry, and Light Detection and Ranging (LiDAR), are widely applied in stream and river habitat monitoring nowadays (Dietrich, 2016; Marcus et al., 2003; Tompalski et al., 2017). For instance, satellite-derived land cover metrics have been employed to rank stream habitat health (Snyder et al., 2005), and a combination of LiDAR and aerial photogrammetry has been developed to accurately delineate riparian terrains and monitor floodplain morphology changes (Lallias-Tacon et al., 2017). Nevertheless, these techniques face notable constraints. Spatial resolutions of remote sensing data, such as 30 m for LandSat 8 and 10 m for Sentinel-2, are challenging to capture fine-scale in-stream and riparian habitat features (Marcus & Fonstad, 2008; Nagendra et al., 2013). Their revisit frequencies, often weeks to months apart, make it difficult to collect up-to-date information for rapid habitat changes caused by extreme weather events (Wulder et al., 2015). Furthermore, the high cost of advanced techniques, such as airborne LiDAR, and the challenge of integrating data from multiple surveys further limit their practicality for achieving the required data quality (Okyay et al., 2019). To date, developing cost-effective and efficient remote sensing methods for stream habitat monitoring remains an ongoing challenge.

In recent years, drone-based surveying technologies, particularly unmanned aerial vehicles (UAVs), have rapidly advanced and been increasingly applied to monitor stream habitat health (Flener et al., 2013; Langhammer, 2019). This growth is driven by several factors. Advances in optical sensors, hovering stabilization, and GPS integration now enable UAVs to capture high-resolution 4 K images or 1080P videos with precise flight paths. Additionally, improvements in drone flight control applications, featuring autopilot capabilities for tasks such as takeoff, route-following, image capture, and landing, allow for fully automated operation via mobile devices (Terry et al., 2021). These capabilities make UAVs highly flexible for frequent deployment—often within hours or days after disturbances—enabling near-real-time assessment of habitat changes and continuous tracking of subsequent restoration progress. Moreover, image processing algorithms, such as Structure from Motion (SfM), can reconstruct high-resolution three-dimensional terrains with mean horizontal errors below 0.1 m and vertical errors under 0.3 m (Elkhrachy, 2021; Turner et al., 2013). Despite these technological advancements, the application of UAVs for stream habitat health evaluation remains rare. To date, there are no integrated UAV tools for multi-metric stream habitat health evaluation, as far as the authors are aware.

This study addresses this gap by developing a suite of cost-effective UAV-based toolkits that automate flight planning, image reconstruction, and quantitative MMI-based habitat assessment. Their effectiveness is demonstrated through application to a case study stream. The paper is structured as follows: the “Methods” section describes the study site, the “Methods” section details the methodology for flight missions, image processing, and habitat health assessment, Sect. 4 presents the Results, the “Discussion” section discusses potential errors, alternative depth measurements, toolkit cost-effectiveness, and toolkit generalization, and the “Conclusions” section provides conclusions.

## Methods

### Study site

**Fig. 1:**
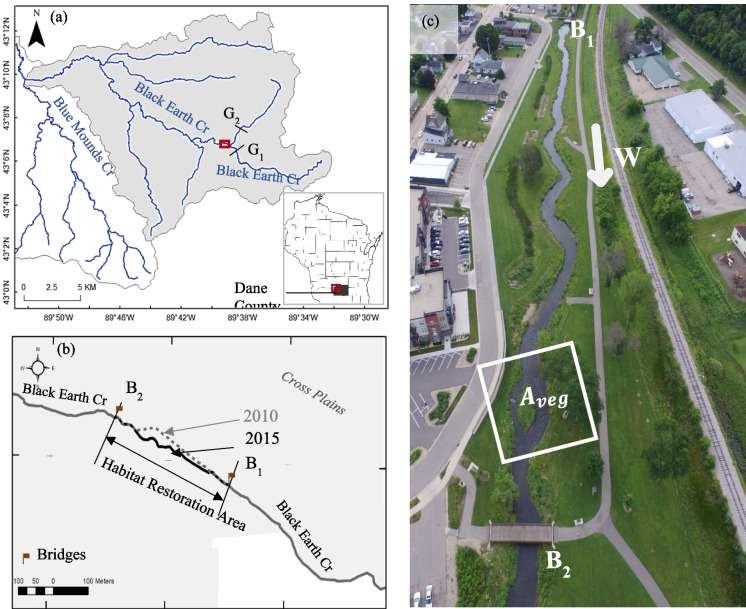
Study area of Black Earth Creek in Dane County, WI. (**a**) Black Earth Creek Watershed. G_1_ and G_2_ are the two USGS stream gauges. (**b**) The 2010 straightened stream (dashed line) and the 2015 meandered stream (solid line) channel. B_1_ and B_2_ are the upstream and downstream bridges, respectively. (**c**) An oblique aerial photo of the study site. The symbol “*W”* represents the flowing direction of Black Earth creek toward the west. The vegetated riparian area, *A*_*veg*_*,* is shown as a white solid rectangle

The study site is the area between bridges B_1_ and B_2_ at the headwater of Black Earth Creek in the village of Cross Plains, Wisconsin (see Fig. 1). Black Earth Creek, a high-quality trout stream, is a 27-mile-long tributary flowing westward to Blue Mounds Creek, Dane County, WI in Fig. 1a. The main creek has an average width and water depth of 8.6 m and 1.4 m, respectively (Wisconsin Department of Natural Resources, 2019). The base flow is approximately 0.8 m^3^/s, based upon the two upstream gauges (USGS 05406457 and 05406469), denoted as G_1_ and G_2_. The stream is classified as a Class 1 type with sufficient natural reproduction to sustain wild trout populations (Wisconsin Department of Natural Resources, 2017). The original straight channel between B_1_ and B_2_ (the dashed line in Fig. 1b) was restored in 2014. The meandering stream, shown as the solid line in Fig. 1b, with riparian grasses and a sequence of riffles and pools in Fig. 1c built to enhance fish habitat (Wisconsin Department of Natural Resources, 2019). In the summer of 2018, an extreme rainfall storm event yielded a historically high flow rate of 29.73 m^3^/s that surpassed the previously recorded water level and incurred severe floods. One vegetated riparian area_,_ shown as a dashed rectangle *A*_*veg*_ in Fig. 1c, experienced degradation and severe erosion.

### Development of toolkits

**Fig. 2:**
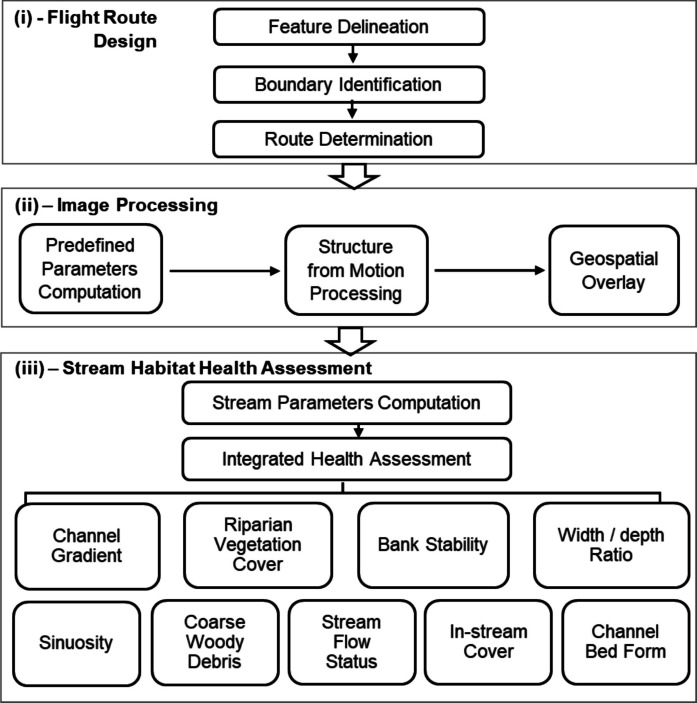
Toolkit Flowchart. (i) Toolkit I as flight route design, (ii) Toolkit II as image processing, and (iii) Toolkit III as stream habitat health assessment

The toolkits were developed as a suite of Python scripts designed to streamline three key tasks: flight route planning for stream surveys, image processing for generating terrain maps and digital elevation models (DEMs), and MMI computation for evaluating stream habitat health, as illustrated in Fig. 2. In contrast to conventional UAV–SfM workflows that typically end with qualitative image interpretation, the proposed framework integrates spatially overlaid high- and low-elevation imagery, autopilot-based flight planning, and MMI assessment into a unified pipeline, reducing manual effort and enabling quantitative, repeatable stream-habitat evaluation.

#### Flight route design

**Fig. 3:**
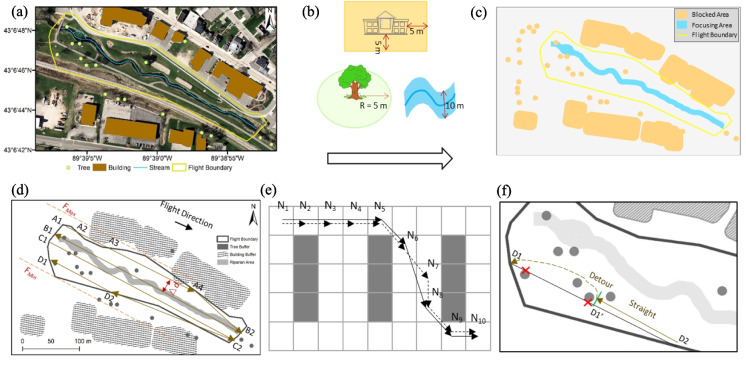
Illustration for Toolkit I. (**a**) Marked trees, buildings, the stream channel and flying boundary. (**b**) Buffer distances for blocked buildings, trees, and riparian focused area. (**c**) Overlaid sketch of blocked areas, focusing areas, and flight boundaries. (**d**) An example of a high-elevation flying route in an S-shape pattern. (**e**) An example of a low-elevation flying route by the Modified D-Lite algorithm. (**f**) The straight route blocked by obstacles (solid line) and the detoured route after the MDL algorithm (dashed line from D1’ to D1)

Toolkit I, shown in Fig. 2i, optimizes flight routes for stream surveys by minimizing flying time, avoiding obstacles, and achieving desired image resolution. First, feature delineation involves digitizing georeferenced aerial maps using ArcMap 10.8 to classify features such as trees, buildings, streams, and flight areas (Fig. 3a). Second, boundary identification processes these features to establish buffer zones for obstacle avoidance and focusing areas that require greater attention. Buffer distances are set to 5 m for trees and buildings, while a 10 m from stream channel is set as the focusing area (Fig. 3b–c). Third, route determination generates two optimized flight routes for high-elevation (30 m above ground) and low-elevation (5 m above ground) surveys. The high-elevation route covers the overall study site, as illustrated in Fig. 3d. The routes are connected in an S-shaped pattern (e.g., A_1_ → A_2_ → A_3_ → A_4_ → B_2_ → B_1_ →C_1_ → C_2_ → D_2_ → D_1_). The interval between parallel flight boundaries (the two red lines, denoted as *F*_*max*_ and *F*_*min*_) is set to ∆d to achieve at least a 75% image overlap, with a 90% overlap applied in focused areas. The low-elevation route addresses areas blocked or shaded by canopy in the high-elevation route. The Modified D-Lite (MDL) algorithm (Koenig & Likhachev, 2002; Ramalingam & Reps, 1996) is employed to generate detoured paths that avoid obstacles. Detailed MDL steps are provided in Supplementary-Table 1. Figure 3e demonstrates an example of an original route (N_1_ → N_2_ → N_3_ → N_4_ → N_5_ → N_6_ → N_7_ → N_8_ → N_9_ → N_10_). After applying the MDL algorithm, redundant nodes along the same straight line (e.g., node N_2_, N_3_, and N_4_) and unnecessary turning nodes that do not influence obstacle avoidance (such as N_7_) are removed, resulting in a smoother detoured route (N_1_ → N_5_ → N_6_ → N_8_ → N_9_ → N_10_). Figure 3f compares the two routes: the original straight route blocked by obstacles (solid line) and the detoured route after applying the MDL algorithm (dashed line from D_1_’ to D_1_). Finally, the flight route designs are saved as shapefiles for UAV deployment, ensuring efficient and obstacle-free drone operation.

#### Image processing

**Fig. 4:**
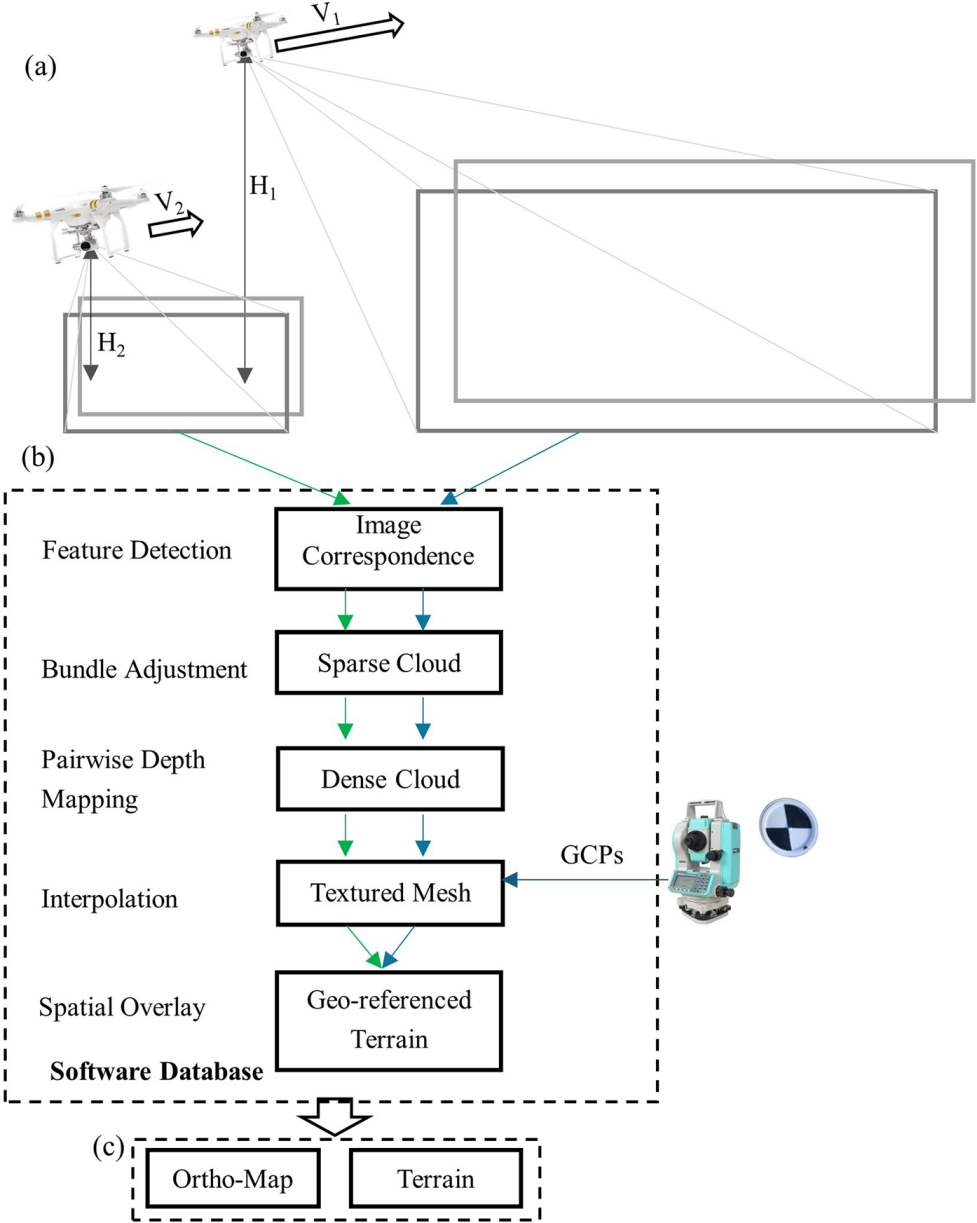
Illustration for Toolkit II. (**a**) Drone images obtained from both high-elevation (H_1_) flying and low-elevation (H_2_) flying routes. (**b**) Five imaging processing steps with drone imagery and in-situ ground control points (GCPs). (**c**) Output: ortho-images and terrain maps

Toolkit II, as shown in Fig. 2ii, processes UAV imagery to reconstruct three-dimensional georeferenced terrains using Agisoft Metashape, chosen for its compatibility with the Python application programming interface (API). To optimize processing time, the toolkit applies predefined quality parameters (lowest, low, medium, high, ultra-high) based on two factors: computer hardware settings (e.g., CPU cores, frequency, GPU cores) and memory requirements determined by image number, size, and quality (Supplementary-Table 2 and Supplementary-Table 3). Figure 4 depicts the workflow for Structure from Motion (SfM) and geospatial overlay, where imagery from high- and low-elevation flights (Fig. 4a) is processed in separate pipelines, represented by brown arrows for high-elevation and green arrows for low-elevation images (Fig. 4b). The five key processing steps include feature detection to align images, bundle adjustment to identify drone camera positions and create sparse point clouds, pairwise depth mapping to generate dense point clouds, geospatial interpolation and color blending to produce textured terrain surfaces, and spatial overlay to merge high- and low-elevation surfaces into georeferenced terrain maps. Predefined parameters are applied at each step to optimize efficiency, and the final outputs are ortho-images and terrain maps (Fig. 4c).

#### Stream habitat health assessment

**Fig. 5:**
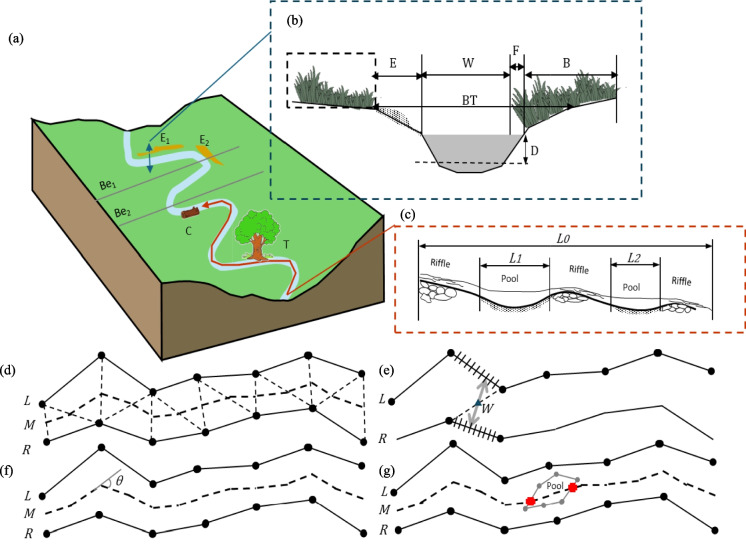
Illustration for Toolkit III: stream habitat indices definition. (**a**) Schematic of a meandered stream with eroded banks (E_1_, E_2_), coarse wood debris C, bends (Be_1_, Be_2_), and tree T. (**b**) Definition of eroded bank width E, stream width W, fish cover width F, buffer width B, bank top width BT, and average stream depth D. (**c**) Definition of pool length. (**d**) Stream length calculation using the MAT algorithm. (**e**) Stream width calculation for a median axis point. (**f**) Bend identification. (**g**) Pool length calculation

Toolkit III, illustrated in Fig. 2iii, computes MMIs to perform an integrative stream health assessment. The first step involves selecting relevant parameters based upon the definitions in the Wisconsin DNR guidelines (Wisconsin Department of Natural Resources, 2018), USDA guidelines (Simonson, 1994) and EPA reports (Somerville, 2010; Stevenson & Bahls, 2002). Figure 5a shows the schematic of a stream with various features such as bends (e.g., Be_1_ and Be_2_), erosion areas (e.g., E_1_ and E_2_), trees (T), and coarse woody debris (C). For each transect (Fig. 5b), we calculate a set of geometry parameters including stream width (W), eroded bank width (E), vegetation buffer width (B), fish cover width (F), bank top width (BT) between the left and right banks, and stream depth (D). Along the stream (Fig. 5c), additional parameters such as the meandered stream length (*L*_*0*_) and pool lengths (e.g., *L*_*1*_ and *L*_*2*_) are measured. These parameters are delineated from orthomaps generated by Toolkit II: stream banks represent the land–water interface, eroded zones are areas of bare soil or recessed vegetation, in-stream cover includes emergent vegetation near the land–water interface, and vegetation buffer zones are undisturbed vegetated areas adjacent to the stream. Once all geometry parameters were delineated, the medial axis transformation (MAT) algorithm (Lee, 1982; McAllister & Snoeyink, 2000) is applied to construct the stream centerline C, as depicted in Fig. 5d. The stream width W at a centerline node is determined as the shortest segment length connecting the left (L) and right (R) banks through the node, as shown in Fig. 5e. The erosion width E, fish cover width F, and vegetation buffer width B can be obtained following similar ways. The bank top width (BT) is the distance between the left and right bank tops. Average stream depth (D) at each transect is estimated as the difference between the water surface elevation derived from the terrain map and the riverbed elevation obtained through field measurements. Bend locations are identified as points along the centerline (M) where the turning angle (*θ*) exceeds 30° (Fig. 5f). Pool lengths are computed by intersecting pool area polygons with centerline (M) based on three thresholds: an average stream depth above 70% of transects, an estimated velocity below 70% of transects, and a percentage of white surface water pixels below 10% (Fig. 5g).

The integrated stream health is then assessed based upon the MMIs with 9 parameters out of 67 stream habitat assessment protocols (Somerville, 2010), based on their frequent usage and feasibility of assessment via UAV without additional sensors. Table 1 lists the 9 MMIs: channel gradient M_1_; riparian vegetation buffer M_2_; bank stability M_3_; width/depth ratio M_4_; sinuosity M_5_; stream flow status M_6_; coarse woody debris M_7_; in-stream cover M_8_; and channel bed forms M_9_. Detailed definitions and assessment criteria of each metric can refer to Simonson (1994), Somerville (2010), and Wisconsin Department of Natural Resources (2018). Except for the index M_1_ that is classified as a high- or mild-gradient stream, the rest of indices (i.e., from M_2_ to M_9_) are scored as 10, 7.5, 5, and 2.5 for excellent, good, fair, and poor health conditions, respectively. The integrated assessment is by summing up the scores of scored indices. The overall stream habitat health status is identified as excellent if the total score is greater than 60, good if greater than 45, fair if greater than 30, and poor if less than 30.

**Table 1:** Multi-metric indices (MMIs) for stream habitat health assessment

Indices				Score			
Code	Name	Description		Excellent	Good	Fair	Poor
				10	7.5	5	2.5
M_1_	Channel Gradient^1^	The change of channel bed elevation per unit length (m/m)^2^		–	–	–	–
M_2_	Riparian Vegetation Buffer	The width of riparian zone covered by continuous and undisturbed natural lands (m)		$\ge 10$	$[\mathrm{5,10})$	$[\mathrm{1,5})$	$<1$
M_3_	Bank Stability	The average width of stream bank has been eroded or has the erosion potential (m)		$<0.2$	$[\mathrm{0.2,0.5})$	$[\mathrm{0.5,1})$	$\ge 1$
M_4_	Width/depth Ratio	The ratio of average stream width to the average depth (m/m)		$<7:1$	$[7:\mathrm{1,15}:1)$	$[15:\mathrm{1,25}:1)$	$\ge 25:1$
M_5_	Sinuosity	H	Ratio of the distance between riffles to the stream width (m/m)	$<7:1$	$[7:\mathrm{1,15}:1)$	$[15:\mathrm{1,25}:1)$	$\ge 25:1$
		M	The ratio of meandered channel length to straight line (m/m)	$>3:1$	$(2:\mathrm{1,3}:1]$	$(1:\mathrm{1,2}:1]$	1:1
M_6_	Stream Flow Status	The ratio of stream channel saturated with water (%)		100	$[\mathrm{75,100})$	$[\mathrm{25,75})$	$[\mathrm{0,25})$
M_7_	Coarse Woody Debris	H	The ratio of stream banks covered by trees, logs, and branches (%)	$\ge 70$	$[\mathrm{40,70})$	$[\mathrm{20,40})$	$<20$
		M		$\ge 50$	$[\mathrm{30,50})$	$[\mathrm{10,30})$	$<10$
M_8_	In-stream Cover	The percentage of stream surface water having overtop fish shelter (%)		$\ge 12$	$[\mathrm{7,12})$	$[\mathrm{2,7})$	$<2$
M_9_	Channel Bed Forms	The percentage of stream length with pools (%)		$(\mathrm{40,60})$	$(\mathrm{30,40}]$ $[\mathrm{60,70})$	$(\mathrm{10,30}]$ $[\mathrm{70,90})$	$[0,10][90,100]$
Total Score				$\ge 60$	$[\mathrm{45,60})$	$[\mathrm{30,45})$	$<30$

1. Channel gradient determines the channel category – high gradient channel (H) if M1 value > 0.02, mild gradient channel (M) otherwise

### Field measurements

Drone flight missions and an in situ field survey were conducted in September 2019. The drone mission consisted of a high-elevation route covering the entire study site and a low-elevation route focused on a severely eroded vegetated riparian area, flown with a DJI Phantom 3 Professional (image size: 4000 × 3000) and shown as the dashed polygon in Supplementary-Fig. 1a. The in situ field survey provided independent elevation measurements and transect-based habitat health assessments to validate results from the toolkits, 46 ground control points (GCPs, yellow dots in Supplementary-Fig. 1a) were measured using a Nikon Nivo total station instrument, which has a stated accuracy of 3 mm ± 2 ppm. Two types of GCPs were employed: easily identifiable points visible from an aerial view, such as building corners, and points marked with 1-inch plates for those less distinguishable in aerial imagery. For each GCP, the *x*, *y*, and *z* corresponding to the instrument point (IP) were recorded. The *z*-coordinate data were transformed into elevation values using the North American Vertical Datum of 1988 (NAVD 88), with reference elevations (B_1_ and B_2_) derived from a 2017 LiDAR survey by Dane County, Wisconsin. Among the 46 GCPs, 25 were used for calibration and 21 for validation via RMSE comparison between reconstructed and measured elevations. For the transect-based health assessment, Supplementary-Fig. 1b shows the 14 surveyed transects across the study site. The first transect was conducted downstream of bridge B_1_, with the subsequent 13 transects spaced equally at intervals of *ΔL* until reaching bridge B_2_ (Supplementary-Fig. 1c). At each transect, stream width (W), vegetated buffer width (B), in-stream fish cover (F), bank top width (BT), and erosion width (E) were measured using measuring tapes. Additionally, coarse woody debris (C), riffle-pool sequences, tree shadows (T), and the number of bends (Be) were surveyed and recorded. Stream depth (D) was measured at five equally spaced points within each transect (Supplementary-Fig. 1 d), and the average of these measurements was taken as the mean stream depth.

## Results

### Performance of flight routes

The designed flight routes were evaluated for obstacle avoidance capability and flight time. Supplementary-Fig. 2 illustrates the low-elevation route, where the original S-shaped flight path with 90% overlap would have intersected three obstacles (O_1_, O_2_, and O_3_; black lines in Supplementary-Fig. 2a). The Modified D-Lite (MDL) algorithm successfully avoided these by generating strategic detours (red boxes in Supplementary-Fig. 2b), refining paths near O_2_ and O_3_, and removing 94% of unnecessary nodes (265 to 15) while maintaining a minimum drone-to-tree/building obstacle distance of 5 m. The flight time for the low-elevation flight route is 800.94 s, based upon a 2 m/s flight speed for the transition (i.e., starting and ending) routes and 1 m/s for the S-shape flight route. Adding 30 s for liftoff and landing, the total flight time falls within the allowable range (75%) of the 20-min battery capacity. For the high-elevation route, the total flight time is 697.0 s, based upon an 8 m/s flight speed for the transition segments and a 4 m/s flight speed for the S-shape flight route. The time is also within the allowable range of batteries. Overall, the good performance on obstacle avoidance and flight time efficiency demonstrates the capability of Toolkit I.

### Ortho-terrain map

**Fig. 6:**
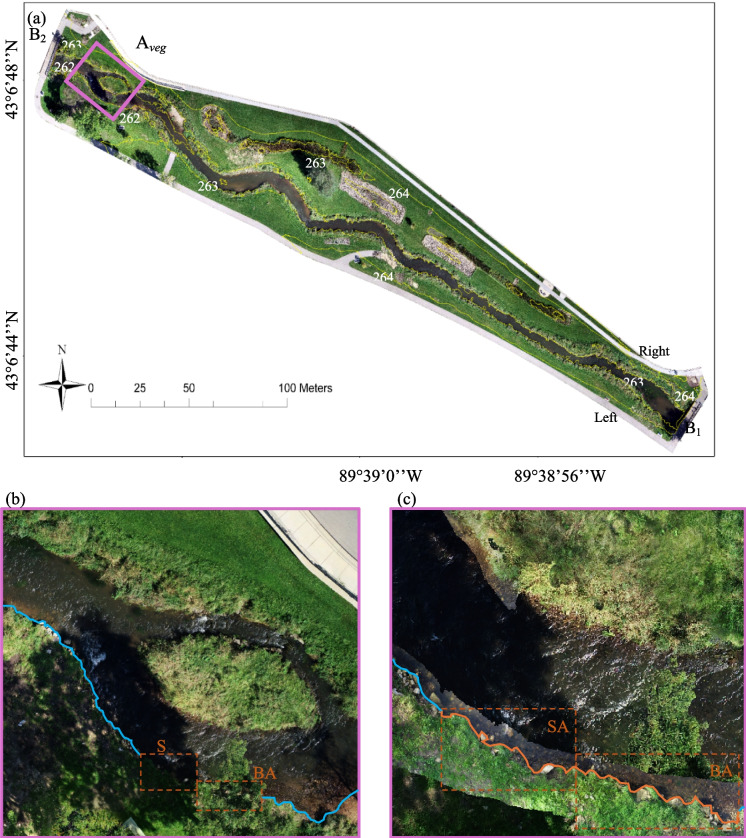
Ortho-Terrain Map Generated from Toolkit II. (**a**) Reconstructed ortho-terrain map combining high- and low-elevation flight missions, with yellow lines marking ground elevation contours and the pink area indicating overlaid low-elevation results. (**b**) and (**c**) compare stream bank delineations for high-elevation results and overlaid results in area *A*_*veg*_. The blue solid line are stream banks detectable from high-elevation results, while the orange solid line indicates stream banks in shaded (SA) or blocked (BA) areas, which are only delineable in the overlaid results

Figure 6a presents the ortho-terrain map generated using 253 high-elevation images for the entire site and 488 low-elevation images specifically for *A*_*veg*_, a canopy-blocked area highlighted in pink squares. Structure-from-Motion (SfM) processing was performed at medium quality, requiring 4.7 h for high-elevation and 7.4 h for low-elevation imagery, achieving texture resolutions of 0.008 m and 0.004 m, respectively. Ground elevation is depicted with 1-m interval yellow contour lines. Validation against ground control points (GCPs) shows an RMSE of 0.04 m horizontally and 0.17 m vertically for the entire site (high elevation, excluding *A*_*veg*_). For *A*_*veg*_ (low elevation), RMSE values increase to 0.09 m horizontally and 0.27 m vertically, reflecting limited image overlap near obstacles and lower texture contrast under canopy. Figure 6b and c underscores the critical role of integrating low-elevation imagery to enhance resolution and clarify features in complex areas. The high-elevation-only result (Fig. 6b) fails to delineate shaded or blocked regions (SA, BA), whereas overlaying low-elevation imagery (Fig. 6c) clearly defines key features such as the stream bank (red lines). This integration highlights the significant value of low-elevation imagery in overcoming limitations posed by obstructed views and ensuring accurate terrain mapping in challenging environments.

### Stream habitat parameters

**Fig. 7:**
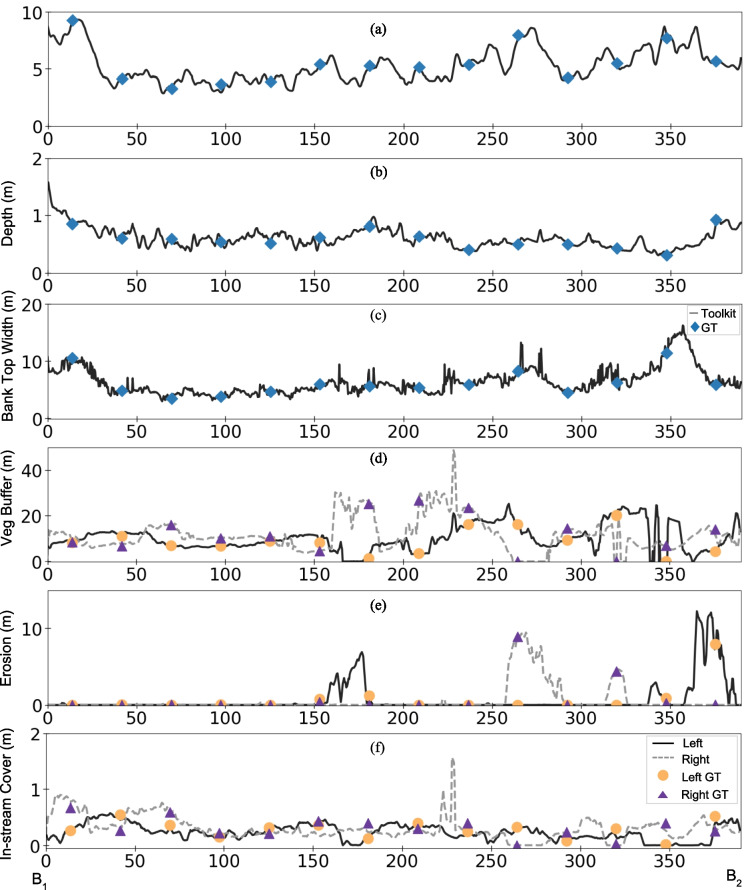
Parameter values for the stream habitat between Bridge B_1_ and B_2_. (**a**)-(**c**) show stream width, water depth, and bank top width (m), with solid lines as toolkit measurements and blue diamonds as ground-truth data for 14 transects. (**d**)-(**f**) present left- and right-bank vegetation buffer width, erosion width, and in-stream cover width (m), with solid and dashed lines for left- and right-bank toolkit outputs and orange/purple markers for ground-truth results for the left and right banks, respectively

Values of stream habitat parameters (concepts illustrated in Fig. 5) are shown in Fig. 7 and Supplementary-Fig. 3. The study examines a 389.59 m stream with an upstream elevation at B_1_ (263.36 m) and downstream elevation at B_2_ (262.09 m). Figure 7 compares continuous drone-based measurements (lines) from our toolkit with transect-based ground truth measurements (blue, orange, and purple markers). The stream width (W) ranges from 2.68 m to 9.50 m after excluding the island width, with the widest section located 14.74 m downstream of B_1_ and the narrowest 64.42 m downstream of B_1_ (Fig. 7a). Water depth (D) varies between 0.27 m and 1.60 m, with the deepest point 1.23 m downstream of B_1_ and the shallowest near the island at 41.38 m upstream of B_2_ (Fig. 7b). Bank top width (BT) ranges from 3.14 m (64.42 m downstream of B_1_) to 15.64 m (32.25 m upstream of B_2_) (Fig. 7c). Vegetative buffer width (B) generally exceeds 1 m, with maximums of 25.19 m (left bank) and 48.74 m (right bank). However, five zones along the banks have no buffer: three on the left bank and two on the right bank (Fig. 7d). Erosion width (E) peaks at 13.05 m (left bank) and 10.74 m (right bank), with significant erosion observed in five specific zones, indicating some local bank instability (Fig. 7e). In-stream fish cover width (F) ranges between 0 m and 0.58 m (left bank) and 0 m to 1.57 m (right bank) (Fig. 7f). Supplementary-Fig. 3 demonstrates the toolkit outputs of pool and bend positions. A total of six pools are characterized, located at 0.11–27.51 m, 31.44–33.46 m, 47.14–49.34 m, 187.41–192.62 m, 276.19–277.31 m, and 284.30–285.85 m, respectively. Additionally, eight bends are identified, located at 66.61 m, 168.52 m, 190.54 m, 225.68 m, 251.62 m, 285.06 m, 331.76 m, and 364.14 m, respectively. The UAV-derived parameters were then compared against tape-based field measurements. The mean absolute difference (MAD) was 0.21 m for stream width (W) and 0.29 m for bank top width (BT). For vegetative buffer width (B), MAD values were 0.57 m and 0.59 m for the left and right banks, respectively, corresponding to relative differences of 6.54% and 4.91%. For erosion width (E), MAD was 0.12 m (left bank) and 0.08 m (right bank), while for in-stream fish cover width (F), MAD was 0.06 m (left bank) and 0.08 m (right bank). Water depth accuracy was assessed using five additional transects located approximately at 50 m, 120 m, 190 m, 260 m, and 330 m along the stream, yielding a mean absolute difference of 0.13 m. Overall, the close agreement between UAV-based and field-based measurements demonstrates the reliability of the proposed toolkits in assessing stream habitat parameters.

### Multi-metric indices (MMIs) assessment

**Fig. 8:**
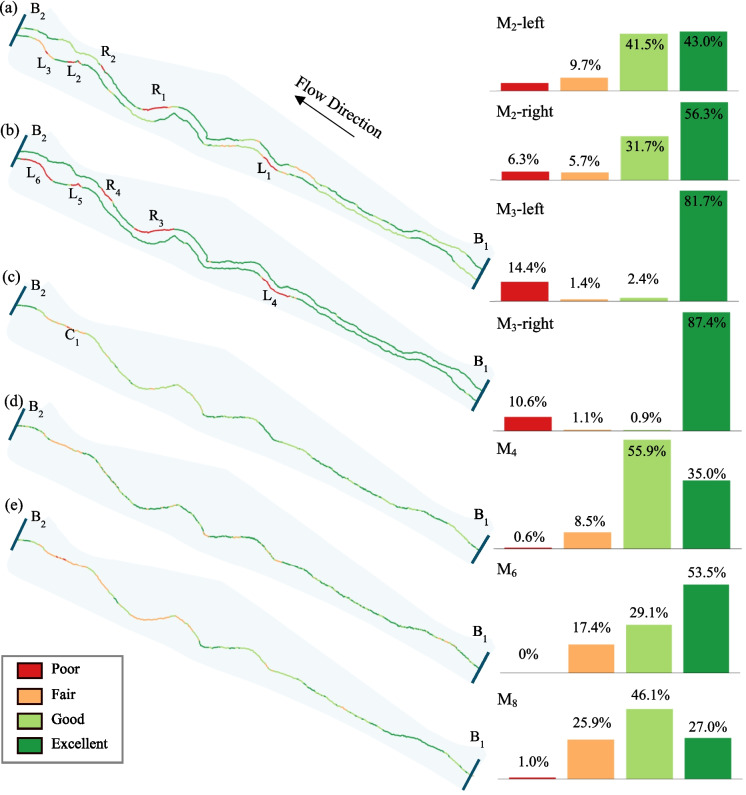
MMI-based stream habitat health assessment (spatially continuous indices: M_2_, M_3_, M_4_, M_6_, and M_8_). (**a**) Visualizes the health condition of M_2_. (**b**) Highlights the health condition of M_3_, (**c**) Displays the distribution of M_4_ health conditions. (**d**) Maps the spatial distribution of M_6_. (**e**) depict the spatial distribution of M_8_. The right column includes histograms for each index, showing the percentages of categories: excellent, good, fair, and poor

MMIs assessments of stream habitat health for the segment between B_1_ and B_2_ are shown in Fig. 8. This segment is characterized as a mild-gradient stream, with an average channel slope (M_1_) of 0.0023 and a maximum slope of 0.0052. Accordingly, the mild-gradient multi-metric system, encompassing M_2_–M_9_ indices, is applied. The riparian vegetation cover metric (M_2_, Fig. 8a) indicates that most stream banks are in excellent or good condition, with undisturbed vegetation buffers exceeding 10 m along 43.0% of the left bank and 56.3% of the right bank. Impaired conditions, spanning 5.8% of the left bank and 6.3% of the right bank (Fig. 8, M_2_-Left and M_2_-Right), are primarily caused by vegetation and soil loss during the 2019 summer flood (e.g., L_2_ and L_3_ on the left bank, and R_1_ and R_2_ on the right bank) or human structures near the stream (e.g., L_1_). The bank stability (Fig. 8b, M_3_) reveals that 81.7% of the left bank and 87.4% of the right bank are stable (Fig. 8, M_3_-Left and M_3_-Right), while poor conditions are observed in zones with material loss (e.g., L_4_ and R_3_) or the presence of bare soil and/or exposed gravel (e.g., L_5_, L_6_, and R_4_). These unvegetated and instable reaches represent priority zones for riparian restoration through native re-vegetation and light bioengineering, which can limit sediment loss, improve shading, and stabilize banks. The width/depth ratio (Fig. 8c, M_4_) shows that over 90% of the stream is classified as excellent or good, except for the downstream segment near B_2_ (C_1_), which features wide and shallow channels. The sinuosity metric (M_5_), with a sinuosity ratio of 1.09:1, is rated fair, as the meandering channel length (389.59 m) is slightly longer than the straight distance between B_1_ and B_2_ (357.98 m). The stream flow status metric (Fig. 8d, M_6_) indicates that 29.1% and 53.5% of the channel are rated as good and excellent, respectively, while 17.4% is under-saturated (i.e., less than 75% water coverage), primarily near the in-stream island. The coarse woody debris metric (M_7_) is poor due to the absence of wood logs, and targeted addition of wood logs along the channel would expand cover habitat for fish and macroinvertebrates. The in-stream cover metric (Fig. 8e, M_8_) shows good or excellent conditions in 46.1% and 27.0% of the stream, respectively, with poor conditions (1%) concentrated near the in-stream island. Lastly, the channel bed form metric (M_9_) is rated as fair, with pools comprising 10.68% of the stream length. Increasing the frequency of riffle–pool sequences through channel re-shaping or wood placements could enhance substrate diversity and create refuge habitats for aquatic fauna.

**Fig. 9:**
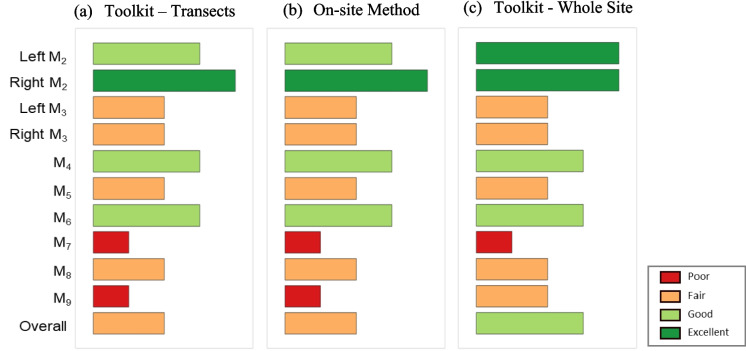
Comparative analysis of stream habitat health assessments using MMIs. (**a**) presents the MMI scores for 14 transects evaluated using our toolkits, (**b**) depicts the MMI scores using on-site methods for the same transects, and (**c**) displays the MMI scores of the entire study site (spatially continuous), obtained using our toolkits

The comparison of habitat health assessment results using our toolkits and in situ transect measurements is shown on Fig. 9. Figure 9a and b presents results from 14 transects, showing consistent health conditions across metrics: excellent for right M_2_, good for left M_2_, M_4_, and M_6_, fair for M_3_, M_5_, and M_8_, and poor for M_7_ and M_9_. The overall MMI score is 43.75 (fair condition) for both the toolkit and in-situ methods, demonstrating the reliability of drone-based assessments. Figure 9c shows integration of continuous results for the entire site, where conditions are excellent for left and right M_2_, good for M_4_ and M_6_, fair for left and right M_3_, M_5_, M_8_, and M_9_, and poor for M_7_. The overall score is 47.5, reflecting good habitat health. While most MMI values match between transects and continuous measurements, transect-based assessments underestimate conditions for left M_2_ (excellent to good) and M_9_ (fair to poor), probably due to uncaptured spatial variations.

## Discussion

### Potential errors

Errors are an inherent aspect of surveying. Although UAV-based assessment tools have demonstrated the capacity to provide accurate evaluations of stream habitats, potential errors may arise from the methodologies employed in Toolkit I, II, and III. Toolkit I: Minor errors can result from misalignments in drone positioning caused by strong winds or weak GPS signals (Wang et al., 2019). These inaccuracies are often exacerbated under tree canopies or near buildings, where GPS signals are obstructed or multipath effects occur. While modern drones equipped with RTK or enhanced GPS systems can reduce such errors, some positional drift may still impact image overlap and route precision, especially in complex riparian settings. Toolkit II: The accuracy of 3D reconstruction depends on the number and quality of tie points among images, which are influenced by processing quality settings (e.g., low to ultra-high) and surface texture. High processing settings improve point-of-interest (POI) extraction in software like Agisoft Metashape but increase computational demand. Homogeneous textures—such as water surfaces, grasslands, tree shadow, and snow—can reduce the number of POIs, limiting effective image matching (Gebrehiwot & Hashemi-Beni, 2021; Turner et al., 2012). These challenges are illustrated in Supplementary-Fig. 4, where water surfaces led to sparse tie-point generation and localized errors in alignment. Toolkit III: Errors primarily stem from streambed variation and in-stream vegetation. Streambed profiles with abrupt changes, such as pools, can be challenging. However, in our study site, this issue is minimal, as pools comprise only a small portion of the streambed between B_1_ and B_2_. Ground truth measurements remain critical for transects with pools, especially in streams with abundant riffle-pool sequences. Additionally, during low-flow periods, tall vegetation may lack sufficient water saturation, requiring supplemental in-situ surveys or airborne humidity sensors for accurate assessment. In summary, while UAV-based toolkits are efficient and accurate for stream habitat assessment, error mitigation through thoughtful planning, quality control, and selective ground validation is essential to ensure robust results.

### Alternative depth measurements

Depth measurements using aerial-based techniques remain challenging due to factors such as stream bottom distortion from reflection (Partama et al., 2018), refraction (Woodget et al., 2019), and water turbidity (Acharya et al., 2021). In this study, water depth was estimated as the elevation difference between the water surface and interpolated streambed profile, which is reliable for the site’s gradually varying bedforms. Several alternative drone-based methods have been proposed for depth estimation. The first applies to dredged channels with regular cross sections (e.g., trapezoidal or parabolic), where Manning’s equation estimates depth under normal-flow conditions. The second uses polarized lens filters to reduce surface reflection (Dolin & Turlaev, 2020), enabling three-dimensional reconstruction of riverbed textures after eliminating glints (Overstreet & Legleiter, 2017). The third infers bathymetry from surface velocity measured via particle image velocimetry (PIV), assuming velocity profiles follow either an exponential function (Hauet et al., 2018) or an entropy relationship (Moramarco et al., 2013) with depth. Moreover, when higher accuracy is required and budget allows, additional drone-mounted sensors—such as topo-bathymetric LiDAR for shallow gravel-bed rivers (Mandlburger et al., 2016) and acoustic profilers for deeper or turbid water (Bandini et al., 2018)—can further improve bathymetric precision.

### Cost-effectiveness of UAV-based toolkits

The cost-effectiveness of the UAV-based monitoring toolkits becomes increasingly evident when applied to tasks involving broad spatial extents, repeated assessments, or complex terrains, where traditional in-situ methods—while long established and valuable—can be labor-intensive, time-consuming, and spatially limited. In our demonstration, the overall investment remained modest: drones with 30-min flight durations and 1080P or 4 K cameras are now commonly available for under USD 1000, and commercial photogrammetry software—such as Agisoft Metashape with basic settings—can be obtained for a few hundred dollars, with open-source alternatives like OpenDroneMap also available. Similarly, open-source software such as QGIS provides comparable functionality for feature digitization and route planning. Once the initial training is completed, the entire workflow from flight planning to habitat assessment can be conducted by a single person. In terms of time cost, surveying a 500-m stream segment typically requires 20–30 min for an S-shaped flight at high elevation. If certain areas are obstructed (e.g., by canopy cover or tall vegetation), low-elevation supplemental flights may be required. For example, collecting low-elevation drone images for areas with similar sizes as *A*_*veg*_ (approximately 25 m × 25 m) requires at most an extra 20 min. Manual delineation of stream features adds one to two hours of indoor efforts, regardless of the number of transects involved. All remaining tasks, such as flight route generation, image processing, terrain reconstruction, and multi-metric index (MMI) assessment, are automated and can run independently without human supervision. As project workload increases—whether through longer reaches, denser transect coverage, or more frequent monitoring—the UAV-based approach becomes increasingly cost-effective while offering high-resolution, spatially continuous outputs. These outputs not only complement but can enhance traditional transect-based methods by revealing habitat degradation patterns or localized impairments that may otherwise go undetected. For water depth estimation, if the streambed profile remains relatively stable over time, depth measurements can be collected once and reused across surveys, further improving efficiency. In cases where bedform changes are expected, alternative depth estimation methods, as discussed in the “Alternative depth measurements” section, can be employed depending on precision needs and budget. Nevertheless, for short stream segments (e.g., < 100 m), one-time assessments, or sites with UAV restrictions, conventional methods may remain more practical. Overall, these UAV-based toolkits are intended not as a replacement, but as a flexible and scalable complement to existing practices—particularly well suited for applications requiring broad spatial coverage, temporal repeatability, and high-resolution data to support informed stream habitat management.

### Toolkit limitation and future generalization

The UAV-based toolkits developed in this study provide a comprehensive approach for assessing stream habitat quality, but several limitations must be acknowledged. First, depth measurements rely on the assumption of a gradually varying riverbed. While this does not affect metric scoring, the absolute depth values are subject to greater uncertainty compared to other physical parameters. Second, the MMI framework was designed following Wisconsin and EPA guidelines for trout streams in non-mountain regions; therefore, its applicability to mountain streams is limited and would require revision. Third, for Index M_3_ (*Bank Stability*), the current approach uses erosion area and width to represent stability conditions. While this method has long been applied in Wisconsin’s trout stream assessments, true bank stability is influenced by multiple factors and may not be fully captured by a single metric. The threshold ranges used here (e.g., < 0.2 m, ≥ 1.0 m) reflect the stream classes and conditions of this study area and may not be directly transferable to other settings. The accuracy of this indicator can also vary with soil properties, channel form, and the viewing geometry of drone imagery. In other environments, more detailed approaches—such as tracking elevation changes over time or estimating soil volume loss and gain—could provide a more robust assessment of stability (Boix-Fayos et al., 2006; Stroosnijder, 2005). These methods would require more ground control points or higher-accuracy drone-mounted GPS to improve elevation precision. Finally, the toolkits focus on physical habitat variables and do not include chemical or biological components, such as water quality, species diversity, or fish spawning areas, which are also critical for comprehensive habitat assessments.

To generalize our toolkits for other study sites or stream habitat health topics, four strategies are proposed. First, the toolkits allow for the exclusion or deemphasis of metrics with significant uncertainty or redundancy, enabling users to prioritize core metrics tailored to specific site conditions. For example, our study site is a restored, meandered channel with sparse vegetation, so metrics such as coarse woody debris are less critical, while vegetation buffer width and in-stream cover are more significant. Second, stream habitat health may exhibit high spatial heterogeneity (White & Walsh, 2020), the toolkits can incorporate vicinity-based assessments by modifying the computation of index values to focus on the poorest conditions within localized areas (*ΔL*/2 of each transect), improving the detection of worst impairments. For instance, at our site, this method identified poor conditions near transects 1, 9, 11, 13, and 14, such as M_2_ and M_8_, which may have been overlooked by traditional methods (see Supplementary-Fig. 5). Third, the toolkits can be expanded to integrate additional indices derived from orthophotos or terrain maps, such as bankfull width and rock and stone embeddedness (Somerville, 2010), using the same processes for parameter computation and health characterization. Finally, the toolkits can be modified to support indices requiring specialized equipment, such as temperature measured with thermal cameras (Kuhn et al., 2021), and water quality detected via multispectral sensors (Kim et al., 2020). By equipping UAVs with portable versions of these instruments, sediment particle size, temperature, and water quality can be processed, assessed, and incorporated into existing MMIs using corresponding function interfaces in our toolkits.

## Conclusions

In this study, a suite of unmanned aerial vehicle (UAV)-based toolkits was developed to evaluate stream habitat health using multi-metric indices (MMIs). The case study conducted at Black Earth Creek, WI, a region with a well-restored stream habitat impaired by a severe flood event, demonstrated the cost-effectiveness and accuracy of the toolkits in assessing MMIs. The toolkits streamline most processes, thereby significantly reducing labor associated with UAV data collection, processing, and assessment. The toolkits comprise three components. The first designs flight routes in a zig-zag pattern, optimizing flight duration while considering image quality, overlap ratio, obstacle avoidance, and smooth detouring. The second component processes UAV imagery to obtain topographic data, including orthophotos and terrain maps, with high texture resolution and accuracy by optimally configuring computer hardware settings. The third quantifies stream habitat parameters and evaluates habitat health using MMIs, yielding results consistent with conventional transect-based ground truth assessments. A key advantage of the toolkits is their ability to provide continuous habitat health evaluations, offering a comprehensive understanding of spatial heterogeneities in stream habitat health. This approach addresses the limitations of transect-only methods, which may overlook critical features between adjacent transects. The toolkits also identify critical hotspots for each metric where habitat degradation is severe. Designed for flexibility and compatibility, the toolkits allow users to adjust metric weights, integrate new metrics from regional protocols, exclude redundant metrics, and embed additional geometrical measurements to accommodate diverse landscapes and habitat types. Given the demonstrated performance, future developments could expand the application to a broader range of stream habitats and integrate them with management and decision-making processes to enhance stream habitat restoration and health.

## Supplementary Information

Below is the link to the electronic supplementary material.

ESM 1 (DOCX 4.62 MB)

## Data Availability

Software: ArcMap 10.8 for feature delineation; Agisoft Metashape 1.7 for terrain map and orthophoto generation. Python scripts: Codes for flight design, processing quality classification, stream parameter computation, and MMI assessments are publicly available at: https://github.com/wwang487/UAVData.git
