# Supplementary material for: Cost-effective drone monitoring and evaluating toolkits for stream habitat health: development and application
Source: Environ Monit Assess. 2025 Dec 5;198(1):10. doi: 10.1007/s10661-025-14814-9 (PMC12680738; doi:10.1007/s10661-025-14814-9)
Supplement: Supplementary file 1 — (DOCX 4.62 MB) [file 10661_2025_14814_MOESM1_ESM.docx]

**Supplementary Information**

**Cost-effective Drone Monitoring and Evaluating Toolkits for Stream Habitat Health: Development and Application**

**Wei Wang *et al*.,**

**Corresponding authors: W. Wang; Email address:** [**wwang487@wisc.edu**](mailto:wwang487@wisc.edu)**.**

**This supplementary document contains three parts.**

**Supplementary Tables: SI-Table1 - SI-Table3**

**Supplementary Figures: SI-Fig.1 - SI-Fig.5**

**Supplementary Tables**

**SI-Table 1**. Modified D-Lite Algorithm for route $S\to T$

| Generate route $R\left( S\to T \right)$ using D-Lite |
| --- |
| Let $N=length(R\left( S\to T \right))$*,* $s_{start}=R\left( 1 \right)$*,* $s_{end}=R\left( 2 \right)$*, res* = [] |
| for $i$ in $3 to N$ |
| if *R*(*i*) in the line $s_{start}\to s_{end}$ |
| then $s_{end}=R\left( i \right)$ |
| else $s_{start}=R\left( i \right)$, *res* = *res* + $s_{start}$ |
| Let $N_{1}=length(res)$, $s_{start}=res\left( 1 \right),$*output* = [] |
| for *j* in 3 to $N_{1}$ |
| if line $s_{start}\to res\left( j \right)$ is blocked or *j* = $N_{1}$ |
| then $output=output+res\left( j \right)$*,* $s_{start}$*= res*(*j*) |
| Return output |

| **Image Quality** | **Image N.O.** | **Estimated Memory Requirement (GB)^1^** | | | | | | | | | | | | | | | |
| --- | --- | --- | --- | --- | --- | --- | --- | --- | --- | --- | --- | --- | --- | --- | --- | --- | --- |
|  |  | **Point Cloud** | **3-D Textured Mesh (Lowest)** | | | **3-D Textured Mesh (Low)** | | | **3-D Textured Mesh (Medium)** | | | **3-D Textured Mesh (High)** | | | **3-D Textured Mesh**  **(Ultra High)** | | |
|  |  |  | Min | Avg | Max | Min | Avg | Max | Min | Avg | Max | Min | Avg | Max | Min | Avg | Max |
| **1080P^2^** | 20 | 0.02 | 0.01 | 0.01 | 0.02 | 0.03 | 0.06 | 0.08 | 0.10 | 0.23 | 0.33 | 0.41 | 0.91 | 1.33 | 1.66 | 3.65 | 5.31 |
|  | 50 | 0.05 | 0.01 | 0.03 | 0.04 | 0.06 | 0.14 | 0.21 | 0.26 | 0.57 | 0.83 | 1.04 | 2.28 | 3.32 | 4.15 | 9.12 | 13.27 |
|  | 100 | 0.50 | 0.03 | 0.06 | 0.08 | 0.13 | 0.29 | 0.41 | 0.52 | 1.14 | 1.66 | 2.07 | 4.56 | 6.64 | 8.29 | 18.25 | 26.54 |
|  | 200 | 1.00 | 0.05 | 0.11 | 0.17 | 0.26 | 0.57 | 0.83 | 1.04 | 2.28 | 3.32 | 4.15 | 9.12 | 13.27 | 16.59 | 36.50 | 53.08 |
|  | 500 | 2.50 | 0.13 | 0.29 | 0.41 | 0.65 | 1.43 | 2.07 | 2.59 | 5.70 | 8.29 | 10.37 | 22.81 | 33.18 | 41.47 | 91.24 | 132.71 |
|  | 1000 | 5.00 | 0.26 | 0.57 | 0.83 | 1.30 | 2.85 | 4.15 | 5.18 | 11.40 | 16.59 | 20.74 | 45.62 | 66.36 | 82.94 | 182.48 | 265.42 |
| **4K^3^** | 20 | 0.02 | 0.02 | 0.05 | 0.07 | 0.11 | 0.23 | 0.33 | 0.44 | 0.91 | 1.33 | 1.77 | 3.65 | 5.31 | 7.08 | 14.60 | 21.23 |
|  | 50 | 0.05 | 0.06 | 0.11 | 0.17 | 0.28 | 0.57 | 0.83 | 1.11 | 2.28 | 3.32 | 4.42 | 9.12 | 13.27 | 17.69 | 36.50 | 53.08 |
|  | 100 | 0.50 | 0.11 | 0.23 | 0.33 | 0.55 | 1.14 | 1.66 | 2.21 | 4.56 | 6.64 | 8.85 | 18.25 | 26.54 | 35.39 | 72.99 | 106.17 |
|  | 200 | 1.00 | 0.22 | 0.46 | 0.66 | 1.11 | 2.28 | 3.32 | 4.42 | 9.12 | 13.27 | 17.69 | 36.50 | 53.08 | 70.78 | 145.98 | 212.34 |
|  | 500 | 2.50 | 0.55 | 1.14 | 1.66 | 2.76 | 5.70 | 8.29 | 11.06 | 22.81 | 33.18 | 44.24 | 91.24 | 132.71 | 176.95 | 364.95 | 530.84 |
|  | 1000 | 5.00 | 1.11 | 2.28 | 3.32 | 5.53 | 11.40 | 16.59 | 22.12 | 45.62 | 66.36 | 88.47 | 182.48 | 265.42 | 353.89 | 729.91 | 1061.68 |
| **8K^4^** | 20 | 0.02 | 0.09 | 0.18 | 0.27 | 0.44 | 0.91 | 1.33 | 1.77 | 3.65 | 5.31 | 7.08 | 14.60 | 21.23 | 28.31 | 58.39 | 84.93 |
|  | 50 | 0.05 | 0.22 | 0.46 | 0.66 | 1.11 | 2.28 | 3.32 | 4.42 | 9.12 | 13.27 | 17.69 | 36.50 | 53.08 | 70.78 | 145.98 | 212.34 |
|  | 100 | 0.50 | 0.44 | 0.91 | 1.33 | 2.21 | 4.56 | 6.64 | 8.85 | 18.25 | 26.54 | 35.39 | 72.99 | 106.17 | 141.56 | 291.96 | 424.67 |
|  | 200 | 1.00 | 0.88 | 1.82 | 2.65 | 4.42 | 9.12 | 13.27 | 17.69 | 36.50 | 53.08 | 70.78 | 145.98 | 212.34 | 283.12 | 583.93 | 849.35 |
|  | 500 | 2.50 | 2.21 | 4.56 | 6.64 | 11.06 | 22.81 | 33.18 | 44.24 | 91.24 | 132.71 | 176.95 | 364.95 | 530.84 | 707.79 | 1459.81 | 2123.37 |
|  | 1000 | 5.00 | 4.42 | 9.12 | 13.27 | 22.12 | 45.62 | 66.36 | 88.47 | 182.48 | 265.42 | 353.89 | 729.91 | 1061.68 | 1415.58 | 2919.63 | 4246.73 |
| Notes: 1. The memory requirement is estimated based on Agisoft support documents, Agisoft community forums (<https://www.agisoft.com/forum/index.php?topic=7434.0>), and third-party evaluations (<https://repository.cimmyt.org/server/api/core/bitstreams/6ba0ff9b-4834-49d6-9395-26d808a16351/content>; <https://www.pugetsystems.com/solutions/photogrammetry-workstations/agisoft-metashape/hardware-recommendations/>). | | | | | | | | | | | | | | | | | |
| 2.Most 1080P UAV-images have size of 1920 × 1080 pixels | | | | | | | | | | | | | | | | | |
| 3.Most 4K UAV-images have sizes of 3840 × 2160 pixels | | | | | | | | | | | | | | | | | |
| 4.Most 8K UAV-images have sizes of 7680 × 4320 pixels | | | | | | | | | | | | | | | | | |

**SI-Table 2.** Estimated memory requirement for point cloud and 3-D texture mesh computation for different image quality conditions

**SI-Table 3.** Suggested Processing Quality for different computer hardware conditions

| **Code** | **CPU Cores** | **CPU Frequency (GHZ)** | **Cuda cores** | **Suggested Processing Quality** | | | | | | |
| --- | --- | --- | --- | --- | --- | --- | --- | --- | --- | --- |
|  |  |  |  | Alignment | Dense Point Cloud | Mesh | | Orthomap | | |
| A | 4 | 2+ | No GPU | Lowest | Lowest | | Lowest | | Lowest |  |
| B | 6 | 3+ | 1000 | Low | Low | | Low | | Low |  |
| C | 8 | 3.5+ | 2000 | Medium | Medium | | Medium | | Medium |  |
| D | 12 | 4+ | 3600 | High | High | | Medium | | Medium |  |
| E | 16 | 4+ | 7200 | High | High | | High | | High |  |
| F | 24 | 4+ | 9600 | Ultra-High | Ultra-High | | High | | High |  |
| G | 32 | 4+ | 10250 | Ultra-High | Ultra-High | | Ultra-High | | Ultra-High |  |

**Supplementary Figures**


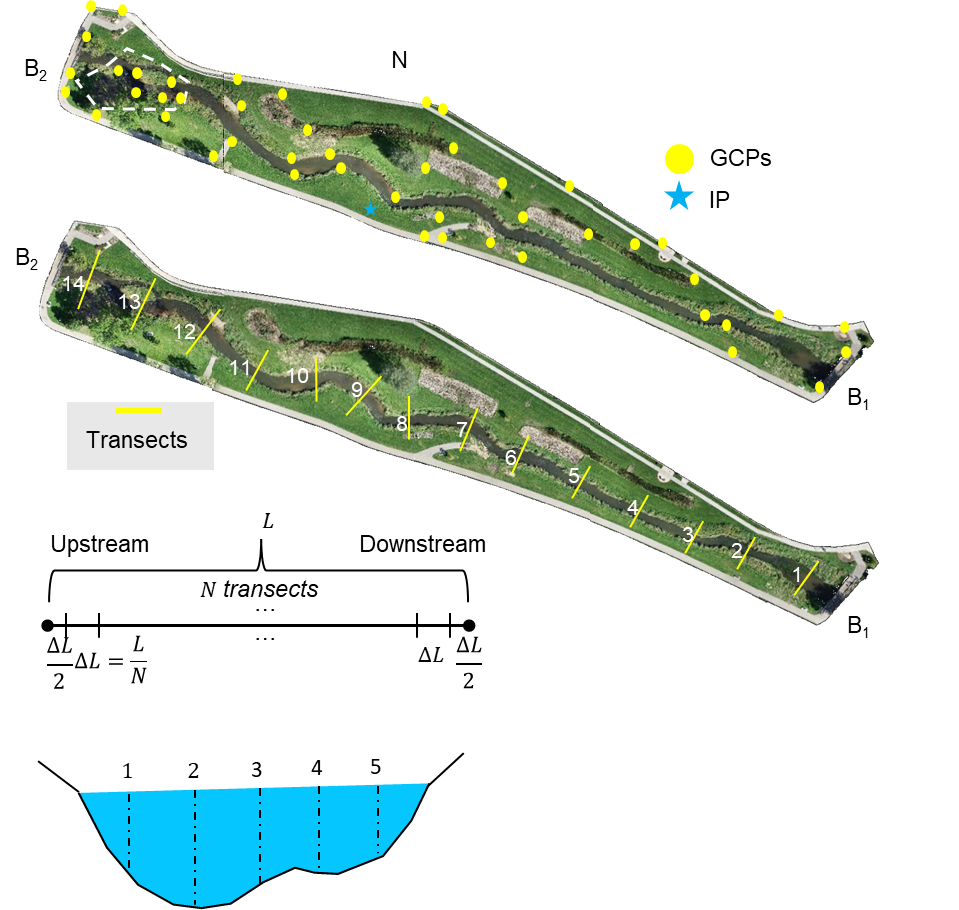


(a)

**SI-Fig. 1. The sampling methods of on-site validation.** (a) Locations of ground control points (GCPs, yellow dots) and the instrument point (IP, blue star); (b) Locations of 14 transects for tape-based measurements; (c) The way to select transects within a stream segment; (d) The way to equally sample within a transect side view.

(b)

(c)

(d)


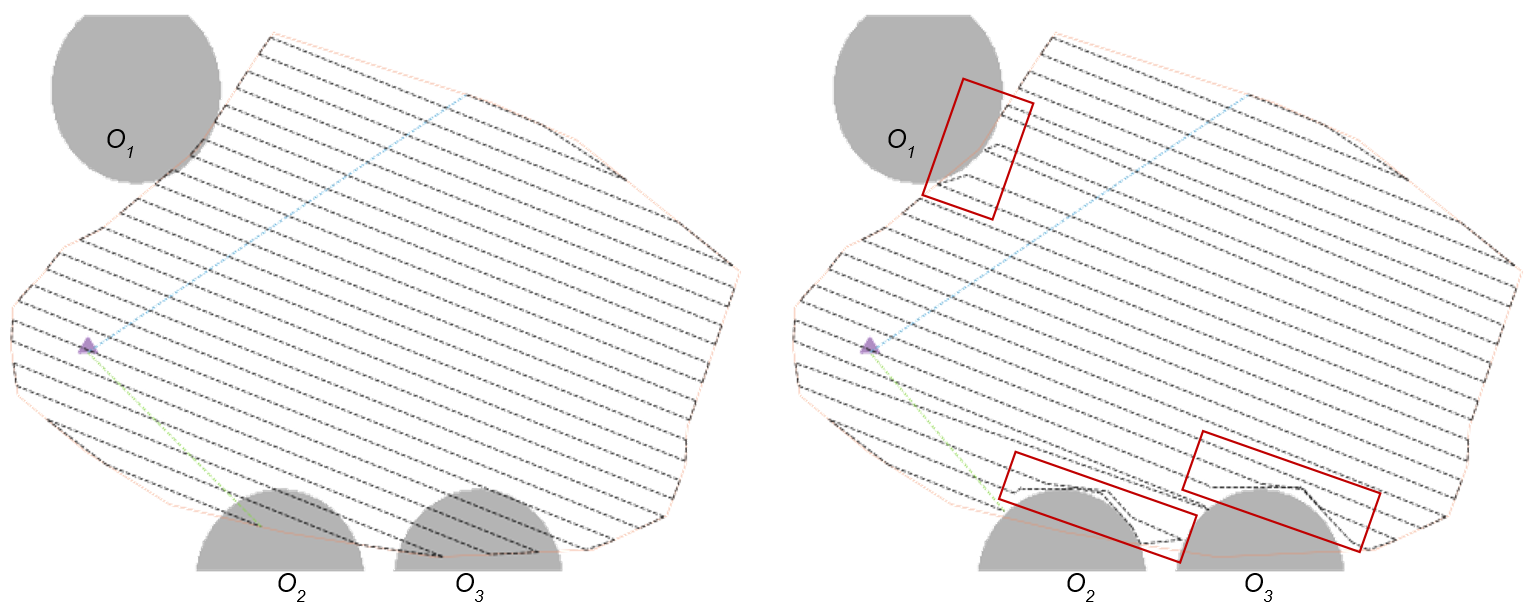


(a)

(b)

**SI-Fig. 2.** **The low-elevation flight routes.** (a) Flight route without obstacle avoidance. (b) Flight route with obstacle avoidance using the Modified D-Lite algorithm. Obstacles are denoted as O_1_, O_2_, and O_3_, while the take-off point is denoted by a purple triangle. The flight boundary is outlined by a thin orange polygon. The S flight route is depicted as a black dashed line with transition routes to the starting point and from the ending point marked as green and purple lines, respectively. The detours from the original route are highlighted by red boxes.

**SI-Fig. 3.** Detected stream bend and pool positions between Bridge B_1_ and B_2_. Eight bends (Bend 1 to Bend 8, marked as orange triangle) and six pools (Pool 1 to Pool 6, marked as dark blue line) are identified.


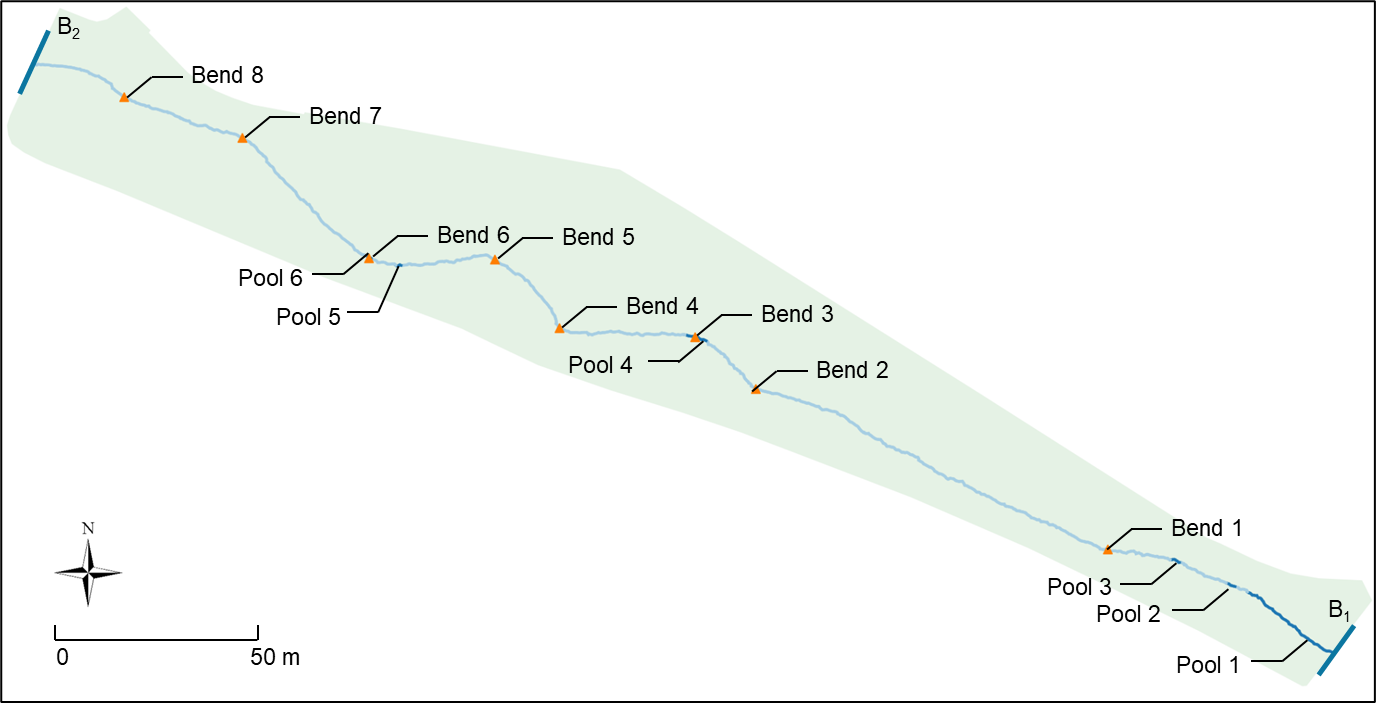

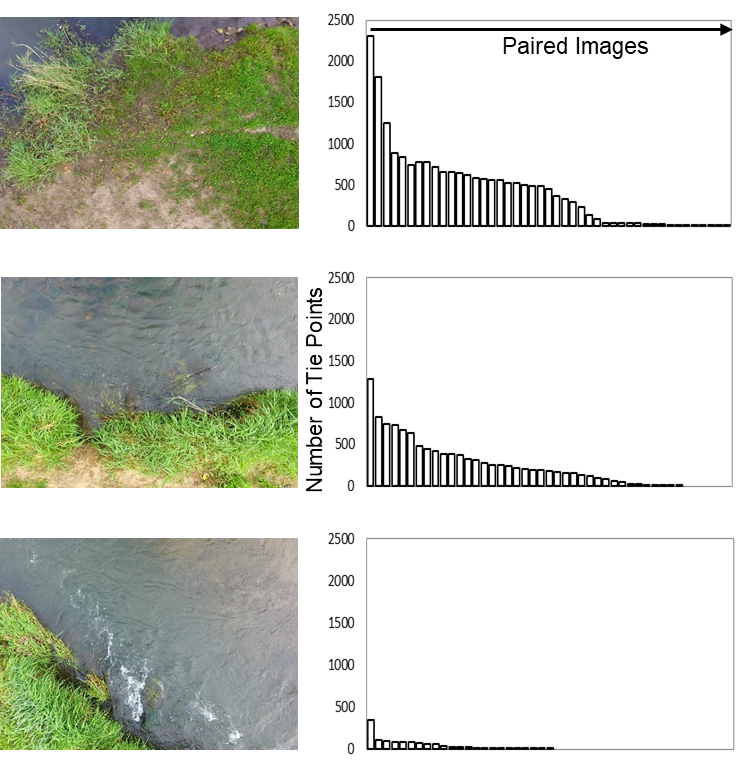


(a)

(b)

(c)

(d)

(e)

(f)

**SI-Fig. 4. The decrease of tied points caused by water surface.** (a) (c) (e) are images with different water body ratio, (b) (d) (f) show the ranked number of tie points we can extract from each image pair.


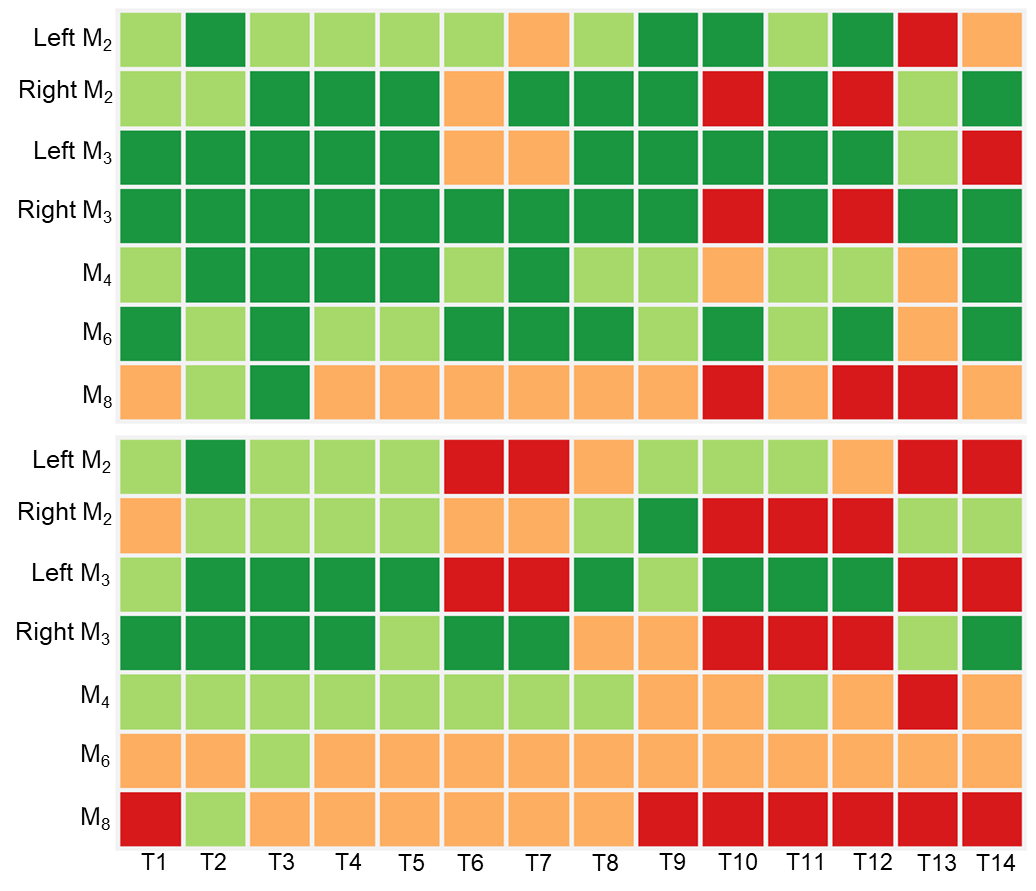


(a)

(b)

**SI-Fig. 5. Spatial variation of stream habitat health condition.** (a) displays the health condition of each index at each transect, (b) indicates the stream segment's poorest health condition category for each index in the vicinity of each transect.
